# In Vitro Activity of a New Ophthalmic Spray Containing Biosecur^®^ Citrus Extract (Oftasecur^®^) Against *Candida auris* and *Candida albicans* and Preformed Biofilm on Contact Lenses

**DOI:** 10.3390/vision9010012

**Published:** 2025-02-07

**Authors:** Antonio Pinna, Matthew Gavino Donadu, Stefano Dore, Rita Serra, Matteo Sacchi, Giacomo Boscia, Aliz Bozó, Renátó Kovács

**Affiliations:** 1 Department of Medicine, Surgery and Pharmacy, University of Sassari, 07100 Sassari, Italy; stefanodore@hotmail.com (S.D.); rita.serra@ymail.com (R.S.); msacchi@uniss.it (M.S.); 2 Ophthalmology Unit, Azienda Ospedaliero-Universitaria di Sassari, 07100 Sassari, Italy; 3 Hospital Pharmacy, Giovani Paolo II Hospital, ASL Gallura, 07026 Olbia, Italy; mdonadu@uniss.it; 4 Department of Translational Biomedicine Neuroscience, “Aldo Moro” University of Bari, 70124 Bari, Italy; bosciagiacomo@gmail.com; 5 Faculty of Medicine, Department of Medical Microbiology, University of Debrecen, 4032 Debrecen, Hungary; bozo.aliz@med.unideb.hu (A.B.); kovacs.renato@med.unideb.hu (R.K.)

**Keywords:** in vitro activity, Biosecur^®^ citrus extract, Oftasecur^®^, *Candida auris*, *Candida albicans*, contact lenses, biofilm

## Abstract

We investigated the in vitro antifungal activity of a new commercial ocular spray containing Biosecur^®^ citrus extract (Oftasecur^®^) against *Candida auris* and *C. albicans* and assessed its activity against preformed *Candida* biofilm on contact lenses and plastic lens cases. The *C. auris* isolate 12 (NCPF 8973) and the SC5314 *C. albicans* wild-type reference strain were used. Oftasecur^®^’s effect on *C. auris* and *C. albicans* planktonic cells (1 × 10^6^ cells/mL) was tested in RPMI-1640 medium. The concentrations tested were 0.39%, 1.56%, 6.25%, 12.5%, and 25%. The living planktonic cell number was obtained using time-kill experiments. Antifungal activity against preformed *C. auris* and *C. albicans* biofilm on etafilcon A and senofilcon A contact lenses and plastic lens cases was also tested. A significant decrease was found in the living cell number of *C. albicans* after 8–48 h in the presence of Oftasecur^®^ concentrations ranging from 6.25% to 25% (*p* < 0.01–0.001). In the *C. auris* experiments, the cell number was significantly decreased after 8 h incubation in 25% Oftasecur^®^ (*p* < 0.05–0.001). Similarly, 12.5% Oftasecur^®^ was effective against preformed *C. auris* and *C. albicans* biofilm on contact lenses and plastic lens cases. The results suggest that the in vitro antifungal activity of Oftasecur^®^ against *C. albicans* and *C. auris* planktonic cells and preformed fungal biofilm on contact lenses and plastic cases is dependent on the concentrations used. Further research is warranted to establish whether Oftasecur^®^ may play a role in the prevention of contact lens-related *Candida* keratitis and other ocular-surface *Candida* infections.

## 1. Introduction

Fungal keratitis is a relatively uncommon but important cause of ocular morbidity [1]. Over the past few decades, there has been an increase in the incidence of keratomycoses worldwide [2]. Although filamentous fungal keratitis is more common in tropical countries, where it is responsible for >50% of all corneal ulcers, yeast infections occur more often in temperate regions [2,3]. In areas with a temperate climate, contact lens use has been reported to be an important predisposing factor for the development of corneal ulcers caused by *Candida* spp. [2,3,4,5].

Flavonoids, a class of natural products with a polyphenolic structure, have several biological activities, such as anti-inflammatory, antioxidant, anti-mutagenic, and anti-carcinogenic properties [6]. Furthermore, flavonoids extracted from citrus peel have a wide spectrum of antimicrobial activity, which has led to the formulation of Biosecur^®^, a water-soluble and alcohol-free hydro-glycerin citrus extract, used in the food processing industry [7,8,9]. In a former study, Biosecur^®^ exhibited a 6-log reduction in *Vibrio vulnificus* at 2% concentration and a 3-log reduction in the same organism at 0.5% [9]. The antibacterial activity of Biosecur^®^ 2% against *V. vulnificus* was shown to be equivalent to that of tetracycline, and its residual activity was found to persist for at least 2.5 h after application [9].

Recently, a new ophthalmic formulation (Oftasecur Ocular Spray^®^, OFFHEALTH S.p.A., Florence, Italy) containing Biosecur^®^ 0.2%, Hypromellose 0.15%, soybean phospholipids (S80) 1%, sodium tetraborate decahydrate, boric acid, sodium chloride, and distilled water became commercially available [10]. This ophthalmic spray delivers droplets to the eyelid margin, thus allowing the drug molecules to mix with the tear film during blinking. Clinical and experimental evidence indicates that Oftasecur^®^ has rapid antimicrobial activity against *Staphyloccocus epidermidis*, *S. aureus*, *Streptococcus pyogenes*, *Pseudomonas aeruginosa*, *Escherichia coli*, and *Candida albicans*, with an optimal tolerability profile [10,11].

The aim of this study was to analyze the in vitro antifungal activity of Oftasecur^®^ against *C. auris* and *C. albicans* and investigate its activity against preformed *C. auris* and *C. albicans* biofilm on contact lenses and plastic contact lens cases. This is an interesting topic, as lid margin antimicrobial sprays would be a useful addition to clinical management, particularly for patients with complex conditions.

## 2. Materials and Methods

### 2.1. Isolates and Growth Conditions

Whole-genome-sequenced *C. auris* isolate 12 (NCPF 8973), obtained from the National Mycology Reference Laboratory, Bristol, UK, was used (accession no.: JANPVY000000000) [12]. The isolate was derived from the South Asian/Indian lineage and showed a non-aggregating phenotype [13]. For the test strain, the MICs were 0.125 mg/L, 1 mg/L, 0.125 mg/L, 4 mg/L, and 0.25 mg/L for anidulafungin, caspofungin, micafungin, fluconazole, and amphotericin B, respectively. The isolate was maintained and cultured on yeast peptone dextrose (YPD) agar (1% yeast extract, 2% mycological peptone, 2% glucose, and 2% agar, at a pH of 5.6).

The SC5314 *C. albicans* wild-type reference strain was used. This strain was sensitive to anidulafungin, caspofungin, micafungin, 5-flucytosine, voriconazole, itraconazole, and fluconazole. The isolate was maintained and cultured on YPD, as reported above.

Susceptibility to Oftasecur^®^ was determined in Roswell Park Memorial Institute-1640 (RPMI-1640) medium (with L-glutamine and without bicarbonate, pH 7.0, with MOPS; Merck, Budapest, Hungary). In the experiments, the commercial formulation of Oftasecur^®^ was serially diluted twofold, so as to reach 25%, 12.5%, 6.25%, 3.125%, 1.56%, 0.78%, and 0.39% of its original concentration, as specified below. The experimental principle was to screen the effect of Oftasecur^®^ through different dilutions. Experiments were performed in duplicate. Oftasecur^®^ was stored at room temperature according to the manufacturer’s instructions.

### 2.2. Contact Lenses

In this study, 1-day Acuvue^®^ Moist with Lacreon^®^ (etafilcon A) and 1-day Acuvue^®^ Oasys with Hydralux^™^ (senofilcon A) with a power of −8.50 diopters were used. Lenses were stored at room temperature according to the manufacturer’s instructions.

### 2.3. Evaluation of Antifungal Activity of Oftasecur Ocular Spray^®^ Against C. auris and C. albicans Planktonic Cells

The effect of Oftasecur^®^ on *C. auris* and *C. albicans* planktonic cells at a concentration of 1 × 10^5^–2 × 10^5^ cells/mL was tested in RPMI-1640 using time-kill experiments at 0.39%, 1.56%, 6.25%, 12.5%, and 25% dilutions. Samples (100 μL) were removed after 0, 4, 8, 12, 24, and 48 h, serially diluted tenfold, plated (4 × 30 μL) onto Sabouraud dextrose agar (Lab M Ltd., Bury, UK), and incubated at 35 °C for 48 h [14]. Positive controls received 1 mL of organisms suspended in sterile phosphate-buffered saline solution (PBSS) at a concentration of 1 × 10^6^ cells/mL, whereas negative control tubes received 1 mL of sterile PBSS. All isolates were tested in two independent experiments, and the mean of the two values was presented. A statistical analysis of the treated and untreated fungal cells was performed by paired Student’s *t*-test using GraphPad Prism 10.2.3. software. The differences between the values for the treated and control cells were considered significant if the *p*-value was <0.05.

### 2.4. Biofilm Formation on Contact Lenses

*C. auris* and *C. albicans* isolates were subcultured on Sabouraud dextrose agar (VWR International Ltd., Debrecen, Hungary). *Candida* cells were harvested by centrifugation (3000× *g* for 5 min) and washed thrice in sterile PBSS. After the final washing, pellets were re-suspended in 5 mL of sterile PBSS and counted using a Burker’s chamber (1 × 10^6^ cells/mL). Contact lenses were washed with sterile PBSS, placed in 12-well tissue culture plates (TPP, Trasadingen, Switzerland) with 4 mL standardized cell suspension (1 × 10^6^ cells/mL), and incubated for 90 min at 250 rpm at 37 °C. Afterwards, non-adherent fungal cells were removed from the lenses by gentle washing with 4 mL sterile PBSS. Then, the contact lenses were immersed in 4 mL sterile RPMI-1640 medium and incubated statically for 24 h at 37 °C [15]. The experiments were performed in duplicate.

### 2.5. Evaluation of Antifungal Activity of Oftasecur Ocular Spray^®^ Against C. auris and C. albicans One-Day-Old Biofilms on Contact Lenses

Based on preliminary experiments, one dilution (12.5%) of Oftasecur^®^ was chosen to evaluate its anti-biofilm effect against one-day-old biofilms over time. Quantitative culturing of sessile cells was performed at 0, 4, 8, 12, 24 and 48 h post exposure. At given time points, the RPMI-1640 medium was removed, and *Candida* preformed biofilms were washed three times with 4 mL of sterile PBSS. Afterwards, the contact lenses were transferred to a centrifuge tube in 4 mL of sterile PBSS, vortexed thoroughly for one minute, and then gently sonicated. Then, aliquots (100 μL) were removed, serially diluted tenfold, plated (4 × 30 μL) onto Sabouraud dextrose agar, and incubated at 35 °C for 48 h [14]. All isolates were tested in two independent experiments, and the mean of the two values was presented. A statistical analysis of the treated and untreated fungal cells was performed by paired Student’s *t*-test using GraphPad Prism 10.2.3. software.

### 2.6. Evaluation of Antifungal Activity of Oftasecur Ocular Spray^®^ Against C. auris and C. albicans One-Day-Old Biofilms Formed on Plastic Contact Lens Cases

Plastic contact lens containers made of polypropylene were used to evaluate the *Candida* one-day-old biofilm’s susceptibility to 12.5% Oftasecur^®^. One-day-old biofilms were developed on the surface of contact lens cases as described above. At given time points (0, 4, 8, 12, 24, and 48 h), the metabolic activity of the biofilms was quantified using a tetrazolium XTT [2,3-bis(2-methoxy-4-nitro-5-sulfophenyl)-2H-tetrazolium-5-carboxanilide] assay, as described previously [14]. Briefly, XTT solution (Merck, Budapest, Hungary) (0.5 g/L) was supplemented with menadione (Merck, Budapest, Hungary) (10 mM prepared in 100% acetone) to a final concentration of 1 μM. The compound was removed prior to the assay of metabolic activity by washing with sterile PBSS. Afterwards, a 100 μL aliquot of XTT/menadione solution was added to each lens case containing the pre-washed biofilms. The plates were covered with aluminum foil and incubated in darkness for 2 h at 37 °C. Following incubation, 80 μL of supernatant from each container was measured photometrically at 492/620 nm [14]. The experiments were performed in duplicate.

In another experimental setting, one-day-old biofilm mass was quantified on the container’s surface at given time points (0, 4, 8, 12, 24, and 48 h) following 12.5% Oftasecur^®^ exposure, as previously published by O’Toole [16]. Briefly, 3 mL of crystal violet 1% was added to each contact lens case containing pre-washed biofilms. These were incubated at room temperature for 15 min. The solution was then removed, and the cases were washed thrice with 3 mL of sterile PBSS to remove unbounded crystal violet. Afterwards, 3 mL of acetic acid 30% was pipetted into each container to solubilize the biofilm-bound crystal violet compounds. After 15 min of incubation at room temperature, 100 μL of supernatant was transferred to a new 96-well plate (TPP, Trasadingen, Switzerland) and read spectrophotometrically at 540/620 nm [16]. The experiments were performed in duplicate. A statistical analysis of the treated and untreated fungal cells was performed by paired Student’s *t*-test using GraphPad Prism 10.2.3. software.

## 3. Results

### 3.1. Evaluation of Antifungal Activity of Oftasecur Ocular Spray^®^ Against C. albicans and C. auris Planktonic Cells

**Figure 1:**
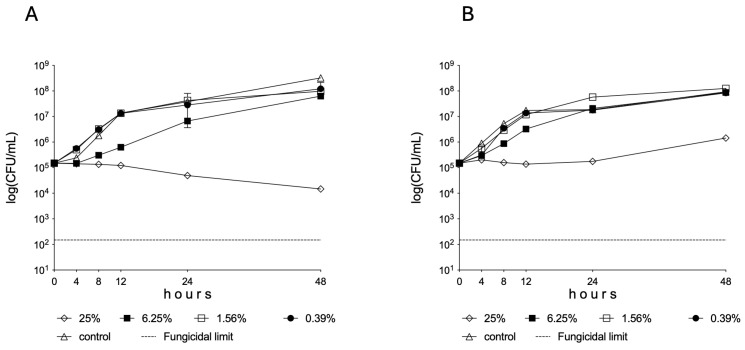
Time-kill curves of 0.39%, 1.56%, 6.25%, and 25% dilutions of Oftasecur Ocular Spray^®^ against *Candida albicans* (**A**) and *C. auris* (**B**) isolates in RPMI-1640 medium. Each time point represents the mean ± standard deviation of the cell count derived from the isolates.

A significant decrease was observed in the living cell number of *C. albicans* between 8 and 48 h in the presence of 25% Oftasecur^®^ dilution (*p* < 0.01–0.001) (Figure 1A). In the *C. auris* experiments, there were essentially static cell counts over time (Figure 1B).

In Figure 1, exact cell numbers are presented. The results obtained at each time point are very close to one another. Therefore, the calculated standard deviation (error bars) cannot be seen properly at certain time points (e.g., Figure 1A at 24 h and Figure 1B at 4 h).

In Figure 1A, a typical concentration-dependent activity can be seen. Indeed, the control values (0% concentration) are lower compared to the treated cell counts. However, these facts could be observed at the lowest concentrations tested. These strange patterns, especially at lower concentrations, are relatively common in the case of time-kill experiments, particularly with agents showing static and not cytocidal effects.

### 3.2. Evaluation of Antifungal Activity of Oftasecur Ocular Spray^®^ Against C. albicans and C. auris One-Day-Old Biofilms Formed on Contact Lenses (Etafilcon A)

**Figure 2:**
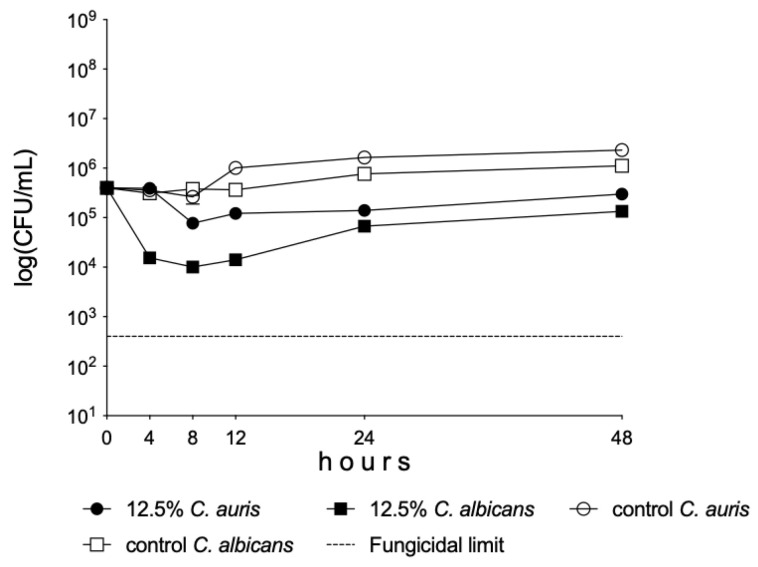
Time-kill curves of 12.5% dilution of Oftasecur Ocular Spray^®^ against *Candida albicans* and *C. auris* isolates in RPMI-1640 medium. Each time point represents the mean ± standard deviation of the cell count derived from the isolates.

A significant decrease was observed in the living cell number of *C. albicans* and *C. auris* between 0 and 24 h in the presence of 12.5% Oftasecur^®^ dilution (*p* < 0.01–0.001) (Figure 2).

In Figure 2, exact cell numbers are presented. The results obtained at each time point are very close to one another. Therefore, the calculated standard deviation (error bars) cannot be seen properly at certain time points (e.g., at 8 h).

Based on preliminary experiments, the 12.5% concentration properly represents the static effect exerted by Oftasecur Ocular Spray^®^ against both *C. albicans* and *C. auris* one-day-old biofilms.

The curves can be seen moving upwards after 24 h. Regrowth of cells or metabolic activity is a natural pattern in the case of agents showing a static effect. It is important to show for how long a given concentration is effective. For example, antifungal activity disappears starting from the time of regrowth.

### 3.3. Evaluation of Antifungal Activity of Oftasecur Ocular Spray^®^ Against C. albicans and C. auris One-Day-Old Biofilms on Contact Lenses (Senofilcon A)

A significant decrease was observed in the living cell number of *C. auris* after 48 h in the presence of 12.5% dilution of Oftasecur^®^ (*p* < 0.05–0.01). Surprisingly, in the case of *C. albicans*, there was no biofilm development observed on the surface of this type of contact lens.

### 3.4. Evaluation of Activity of Oftasecur Ocular Spray^®^ in the Case of the Biofilm Mass of C. albicans and C. auris Sessile Communities Formed on Plastic Contact Lens Cases Using Crystal Violet Assays

**Figure 3:**
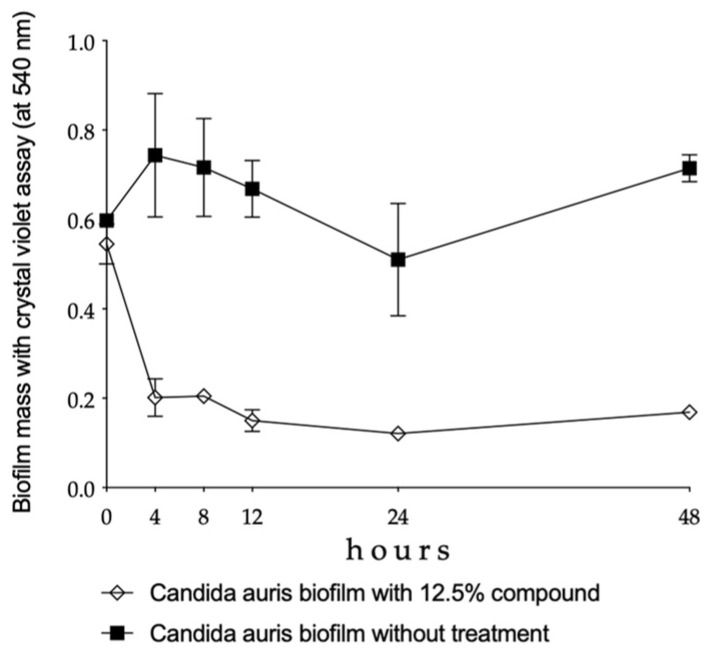
Plot shows changes in the biofilm mass over time in the case of *Candida auris*. Each time point represents the mean ± standard deviation of the biofilm mass derived from the isolates, measured spectrophotometrically at 540 nm.

A significant reduction was observed in the biofilm mass of *C. auris* between 4 and 48 h of being in the presence of the 12.5% Oftasecur^®^ dilution (*p* < 0.01–0.001) (Figure 3).

In Figure 3, the spectrophotometer-derived results are presented. The standard deviation may be naturally wider compared to direct fungal cell-based biofilms due, for example, to more washing steps and the dynamic structure of the biofilms.

Figure 3 shows a decrease after 24 h for the case without treatment. In this case, the biofilm mass changed over time. There might have been a potential washing failure in this experiment, explaining the relatively high standard deviation. There was one outlier value. Nevertheless, this transient decrease did not significantly distort the full pattern of activity.

### 3.5. Evaluation of Activity of Oftasecur Ocular Spray^®^ in the Case of the Metabolic Activity of C. albicans and C. auris One-Day-Old Biofilms Formed on Plastic Contact Lens Cases Using XTT Assays

**Figure 4:**
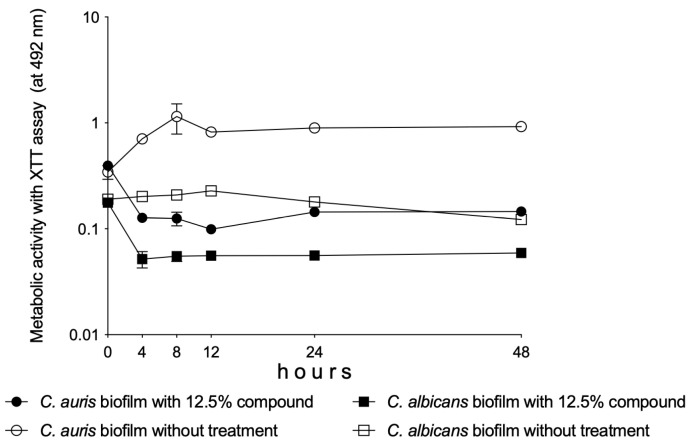
Plot shows changes in the metabolic activity of one-day-old *Candida albicans* and *C. auris* biofilms over time. Each time point represents the mean ± standard deviation of the metabolic activity derived from the isolates, measured spectrophotometrically at 492 nm.

A significant reduction was observed in the metabolic activity of *C. albicans* and *C. auris* biofilms between 4 and 48 h of being in the presence of the 12.5% Oftasecur^®^ dilution (*p* < 0.05–0.001) (Figure 4).

In Figure 4, the spectrophotometer-derived results are presented. The standard deviation may be naturally wider compared to direct fungal cell-based biofilms due, for example, to more washing steps and the dynamic structure of the biofilms.

## 4. Discussion

In this in vitro study, the 25% dilution of a new ophthalmic spray containing 0.2% Biosecur^®^ citrus extract (Oftasecur^®^) was found to have good antifungal activity against planktonic cells of two reference isolates of *C. albicans* and *C auris*. Similarly, the 12.5% dilution of Oftasecur^®^ was effective against preformed *C. auris* and *C. albicans* biofilm on contact lenses and plastic contact lens cases.

In a former investigation, Mencucci and coworkers [11] assessed the in vitro activity of Oftasecur^®^ against *C. albicans* ATCC 10231 and found that it showed good fungicidal activity against this strain, a result consistent with the present study.

*C. albicans* is the most common yeast species associated with mycotic keratitis. The risk factors for *Candida* keratitis include topical steroid use, ocular-surface disease, contact lens wear, previous corneal surgery, and a history of ocular trauma [4,17,18,19,20,21]. Over the past decade, there has been an increase in the incidence of *Candida* keratitis in tropical and nontropical regions. This seems, at least in part, to depend on the increase in contact lens use, in combination with outdoor activities leading to corneal trauma associated with contaminated soil or water [5,21].

Since its first clinical description more than 10 years ago, *C. auris* has emerged as a global public health threat due to its ability to cause nosocomial infections globally in health care environments [22,23]. *C. auris* can colonize a variety of body sites and medical implants, such as indwelling medical devices and contact lenses, with sessile biofilm formation being one of the most important consequences. *C. auris* has high minimum inhibitory concentrations against the three main antifungal groups (azoles, polyenes, and echinocandins). Moreover, the extensive biofilm-forming properties further complicate therapy. As far as we know, only one report exists on keratomycosis caused by *C. auris* [24].

In this study, we performed Oftasecur^®^ susceptibility testing against *C. auris* and compared these findings to those obtained against a reference strain of *C. albicans*.

Although the pathophysiological mechanisms underlying the development of contact lens-related *Candida* keratitis have not been fully elucidated yet, fungal contamination of the ocular surface is thought to play a crucial role [25]. During contact lens use, fungi can gain access to the ocular surface from the environment through contamination of the lens, lens case, and lens care solutions. Several studies on contact lens-associated microbial keratitis have revealed contamination of the care systems [26]. In corneal ulcers associated with contact lens use, microbial contamination of the care systems may represent the source of infection [27,28].

In recent years, the emergence of antifungal-resistant human-pathogenic fungi, including *Candida*, has become an important issue in medicine [29]. The list of antimycotics for the management of *Candida* infection is very limited, while the prevalence of resistant strains is increasing quickly. Therefore, the search for novel antifungal drugs, including disinfectants and natural compounds, is of great importance.

In a former in vitro investigation, a commercially available ophthalmic solution with povidone-iodine (PVP-I) 0.6% as the active ingredient was found to have good, rapid antibacterial activity against multi-resistant isolates of *S. epidermidis*, *S. aureus*, and *P. aeruginosa*, but it was poorly effective against *Candida* isolates [30]. Similarly, PVP-I was found to be less effective against some bacterial spores and fungi [31], but other studies have documented a rapid antifungal activity against clinical strains of *Fusarium* and *Candida* [32,33]. On the whole, these conflicting findings suggest that different strains of *Candida* may show remarkable differences in susceptibility to PVP-I.

Furthermore, a new commercial ophthalmic solution containing hexamidine diisethionate 0.05% has been shown to have in vitro antimycotic activity against *C. albicans*, *C. parapsilosis*, *C. tropicalis*, *C. glabrata*, and *C. krusei* [34].

Oftasecur Ocular Spray^®^, containing a water-soluble and alcohol-free hydro-glycerin citrus extract (Biosecur^®^), showed antifungal activity against *Candida* isolates in our experimental model and in the assays by Mencucci et al. [11].

Fungi can exist as single planktonic cells or form biofilms, aggregations of microorganisms enclosed in a self-produced polymeric matrix and adherent to the surface of tissues and biomaterials [15,35]. Experimental studies have demonstrated that biofilms are far less susceptible to antimicrobials than free-floating planktonic cells [15,36]. *Candida* biofilm has been found on contact lenses and lens cases, and there is increasing evidence that biofilms may play an important role in the development of contact lens-related *Candida* keratitis by allowing organism persistence on contact lens surfaces [15].

In our experimental models, the 12.5% Oftasecur^®^ dilution was found to have a significant effect against *C. albicans* and *C. auris* one-day-old biofilms formed on etafilcon A and senofilcon A contact lenses and plastic contact lens cases. However, it should be noted that the spray was not able to completely eliminate them.

This in vitro study has some limitations. Indeed, different strains of the same species isolated from different sites may show remarkable differences in biofilm production. Therefore, the results cannot be extended to all *Candida* strains. Furthermore, the biofilm formation settings on contact lenses do not fully reflect the clinical environment, which would be better explored in ex vivo models.

In conclusion, the in vitro antifungal activity of Oftasecur^®^ against *C. albicans* and *C. auris* planktonic cells and preformed fungal biofilm on contact lenses and plastic cases was dependent on the concentrations used. Further experimental and clinical research is necessary to establish whether Oftasecur^®^ plays a role in the prevention of contact lens-related *Candida* keratitis and other ocular-surface *Candida* infections.

## Data Availability

The original contributions presented in this study are included in the article. Further inquiries can be directed to the corresponding author.
